# 3D Printing—A “Touch-Button” Approach to Manufacture Microneedles for Transdermal Drug Delivery

**DOI:** 10.3390/pharmaceutics13070924

**Published:** 2021-06-22

**Authors:** Merima Sirbubalo, Amina Tucak, Kenan Muhamedagic, Lamija Hindija, Ognjenka Rahić, Jasmina Hadžiabdić, Ahmet Cekic, Derzija Begic-Hajdarevic, Maida Cohodar Husic, Almir Dervišević, Edina Vranić

**Affiliations:** 1 Department of Pharmaceutical Technology, Faculty of Pharmacy, University of Sarajevo, Zmaja od Bosne 8, 71000 Sarajevo, Bosnia and Herzegovina; merima.sirbubalo@ffsa.unsa.ba (M.S.); amina.tucak@ffsa.unsa.ba (A.T.); lamija.hindija@ffsa.unsa.ba (L.H.); ognjenka.rahic@ffsa.unsa.ba (O.R.); jasmina.hadziabdic@ffsa.unsa.ba (J.H.); 2 Department of Mechanical Production Engineering, Faculty of Mechanical Engineering, University of Sarajevo, Vilsonovo Setaliste 9, 71000 Sarajevo, Bosnia and Herzegovina; kenan.muhamedagic@mef.unsa.ba (K.M.); begic@mef.unsa.ba (D.B.-H.); cohodar@mef.unsa.ba (M.C.H.); 3 Head and Neck Surgery, Clinical Center University of Sarajevo, Bolnička 25, 71000 Sarajevo, Bosnia and Herzegovina; adervisevic@sf.unsa.ba

**Keywords:** microneedles, 3D printing, transdermal drug delivery, printing materials, printing parameters

## Abstract

Microneedles (MNs) represent the concept of attractive, minimally invasive puncture devices of micron-sized dimensions that penetrate the skin painlessly and thus facilitate the transdermal administration of a wide range of active substances. MNs have been manufactured by a variety of production technologies, from a range of materials, but most of these manufacturing methods are time-consuming and expensive for screening new designs and making any modifications. Additive manufacturing (AM) has become one of the most revolutionary tools in the pharmaceutical field, with its unique ability to manufacture personalized dosage forms and patient-specific medical devices such as MNs. This review aims to summarize various 3D printing technologies that can produce MNs from digital models in a single step, including a survey on their benefits and drawbacks. In addition, this paper highlights current research in the field of 3D printed MN-assisted transdermal drug delivery systems and analyzes parameters affecting the mechanical properties of 3D printed MNs. The current regulatory framework associated with 3D printed MNs as well as different methods for the analysis and evaluation of 3D printed MN properties are outlined.

## 1. Introduction

Since the approval of the first transdermal patch containing scopolamine for the treatment of motion sickness four decades ago [1], the transdermal delivery of active pharmaceutical ingredients (APIs) has been proposed as an attractive alternative to parenteral and oral drug delivery. Transdermal drug delivery (TDD) avoids the first-pass metabolism [2], improves drug absorption, and ensures non-invasive, pain-free self-administration compared to the parenteral route, thus improving patient compliance. However, due to the limited transdermal permeability of numerous APIs, chemical or physical enhancers such as electroporation, iontophoresis, jet injection, and sonophoresis were introduced [3]. As these methods were linked to problems such as painful sensations caused by electrodes, deep skin tissue damage caused by high-frequency sonophoresis, etc., the microarray patch technology, which consists of microprojections of different shapes supported on a baseplate, has gained increased attention [4].

Microneedles (MNs) represent skin-friendly puncturing devices of microscale dimensions [5] that are designed to efficiently and painlessly bypass the outermost layer of the skin, the stratum corneum (SC), which acts as a barrier for transdermal penetration of APIs, in particular for those with log p values below 1 and greater than 3 [6], by forming microchannels and thus releasing the drug into the skin’s microcirculation [7,8]. The concept of MNs dates back to the 1970s when Gerstel and Place filled out the patent form (the current assignee is Alza Corporation) [9]. Nearly two decades later, MNs were once again at the center of significant research, highlighting the great advances in microfabrication technology [6].

To meet all requirements for the non-invasive application and efficient drug delivery to the systemic circulation, a successful MN system must be produced by a manufacturing method that guarantees accurate, reproducible, robust, and precise production of MN in the micrometer range [8,10]. To date, manufacturing strategies for MN include techniques such as lithography [11], electrochemical and photochemical etching [12], laser cutting [13], laser ablation [14], metal electroplating [15], laser micromachining, injection molding [16], and micromolding [17]. However, many of these methods involve high production costs, as they require advanced equipment and manual operations, or labor-intensive work that makes it difficult to expand production from the laboratory to the industrial level [7,18]. Consequently, interest has increased in a new accessible and cost-effective manufacturing strategy that fully exploits the potential of TDD by MNs—additive manufacturing (AM)—that appears to be a promising solution [7].

AM, commonly known as three-dimensional (3D) printing, rapid prototyping, or solid free form fabrication (SFF), represents a family of techniques launched in the 1980s that revolutionized not only the pharmaceutical industry [17,19] but also the majority of industrial and scientific fields such as automotive [20], aerospace [21], construction [22], and consumer electronics industries [23]. The term “3D printing” was defined by the International Standard Organization (ISO) as “*the fabrication of objects through the deposition of a material using a print head, nozzle, or another printer technology*” [24]. The reason for the great interest in 3D printing was the possibility for fast, cost-effective, and time-saving prototyping of complex structures with high production rates, reduced material waste, and increased productivity [7,25]. More than 10 different AM technologies have been proposed since Chuck Hull’s first development and commercialization of stereolithography apparatus (SLA) back in 1986. They include material extrusion, vat photopolymerization, material and binder jetting, powder bed fusion (PBF), directed energy deposition, and sheet lamination [26,27,28], which have been used for the precise manufacture of various drug dosage forms for oral [29,30,31], transdermal [26,27,32,33], vaginal [34], and subcutaneous [35] applications, as well as implants and prosthetics, whose high tunability and complexity are unattainable by conventional techniques [7,19]. More recently, the wide range of 3D printing technologies has opened an interesting new field of research to produce MNs, which will be explained in the next sections.

This review provides an overview of the 3D printing technologies used for MN fabrication, including a survey on their benefits and drawbacks. We aimed to highlight current research in the field of 3D printed MN-assisted transdermal drug delivery systems and point out the most important challenges in the commercialization of 3D printed MNs, such as evaluation of MNs, material selection, and the current regulatory framework.

## 2. Microneedles: Characteristics, Classification, and Delivery Strategies

The ultimate success of MN-based drug delivery is founded on the critical parameters, which include their dimensions (shape, size, geometry), manufacturing method, and materials, as well as the type of therapeutics that could be delivered into the skin [10]. MNs are primarily investigated for the possibility of transdermal delivery of small therapeutic molecules [36,37], biomacromolecules [38], hormones [39], peptides [40], vaccines [41,42] (against SARS, MERS, COVID-19), and genes [43], as well as nanoparticles [44] in the treatment of pain [45] (e.g., migraine), and diseases such as diabetes [19,46], or hypertension [47]. It is important to mention that research is being done on the use of MNs in clinical drug monitoring [48]. Just like hypodermal needles, MNs can be used for two-way fluid circulation, allowing extraction of interstitial fluid from the skin [49]. Additionally, the cosmetics industry has shown interest in this technology, as MNs have shown great potential for the treatment of skin imperfections, but also for the delivery of substances to the skin for cosmetic purposes [50,51]. Recent research shows good results in the treatment of eye diseases such as glaucoma using MNs as ocular drug delivery systems [52]. Thus, the functional ability of MNs is well recognized in many research fields, which is confirmed with the fact that more than 250 patents on MNs can be found in databases of the European Patent Office (Munich, Germany) (Espacenet) and the World Intellectual Property Organization (WIPO, Geneva, Switzerland) [53].

**Figure 1:**
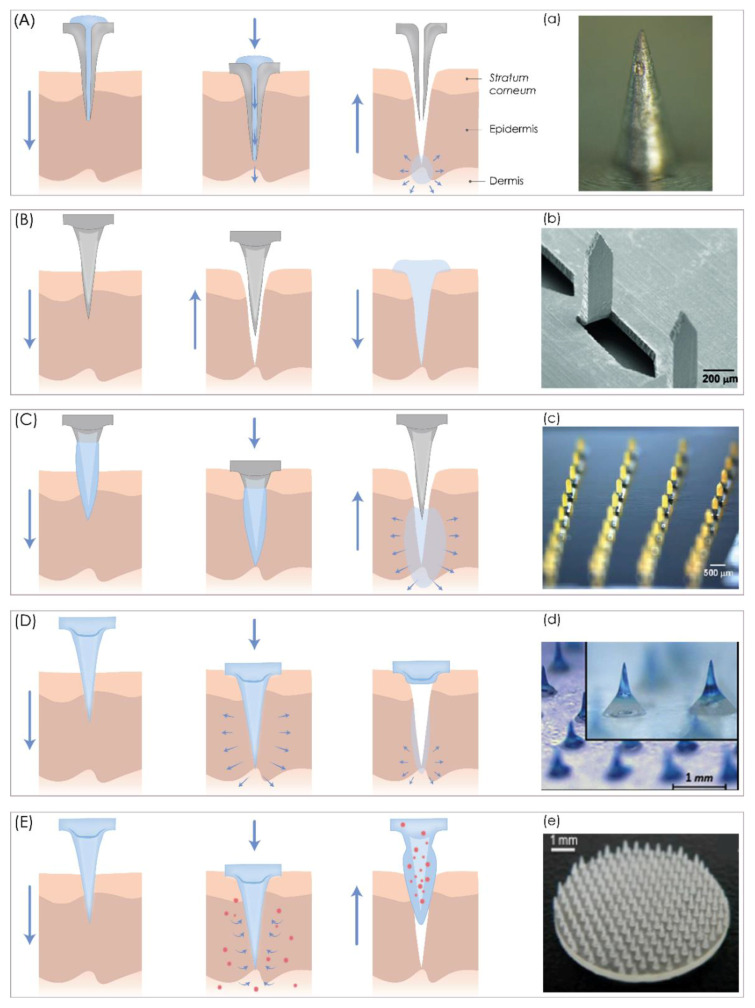
A schematic representation of five different microneedle (MN) types for transdermal drug delivery. (**A**) Hollow MNs puncture the skin and release a liquid drug formulation through the needle lumen. (**B**) Solid MNs create microchannels in the skin and increase drug permeability. (**C**) Coated MNs enable drug dissolution into the skin from the coating film. (**D**) Dissolving MNs release the drug incorporated within the MNs. (**E**) Hydrogel MNs collect interstitial fluids and induce drug release through the swollen microprojections. (**a**) Bright-field microscopy (SZX 16, Olympus, Center Valley, PA, USA) image of hollow MNs. Reproduced with the permission from [80], Springer Nature, 2013. (**b**) SEM microscopy of solid MNs. Reproduced with the permission from [81], Elsevier, Amsterdam, The Netherlands, 2011. (**c**) bright-field microscopy (Olympus SZX12 stereo microscope, Olympus America) image of coated MNs. Reproduced with the permission from [37], Springer Nature, 2007. (**d**) microscope (STC-GE33A, SENTECH, Yokohama, Japan) image of dissolving MNs. Reproduced with the permission from [82], Elsevier, 2013. (**e**) picture of a hydrogel microneedle patch. Reproduced with the permission from [83], John Wiley & Sons—Books, 2015. The image was created with Adobe Illustrator CC (Version 23.0.1.; Adobe Inc., San Jose, CA, USA).

According to their structure and design, MN can be “hollow” or “solid”, but in the context of their application, MNs can be classified into five categories, hollow, solid, coated, dissolving MNs, and swelling MNs, that can be used for both TDD and extraction of interstitial fluids (Figure 1).

Hollow MNs enable a continuous fluid flow of drugs such as insulin [54,55] and vaccines [56] through a 5–70 μm wide lumen or an inner bore in the MNs by a “*poke and flow*” mechanism (Figure 1A) [14,57,58]. They are made from a variety of materials such as glass [55], polymers [59], metal [60], ceramic [61], etc. This type of MN transports drugs across the skin using different methods, such as passive or active diffusion. Unlike the passive diffusion process, the active process requires pressure- or electrically controlled flow of drug solution through a pump, syringe, or pressurized gas [14,60,62,63]. When a microfluidic chip [49,64] or micropump is incorporated with an MN array, a controlled drug release from the drug reservoir can be achieved [65,66]. The main limitations of hollow MNs are the potential of clogging the MN tip during the application and the flow resistance, as the tissue around the MN tips can be compressed [6]. In order to solve the problem of tip clogging, MNs with eccentric holes have been designed [57]. To improve the relatively low infusion rates of 50 to 300 nL/min, hyaluronidase is usually added to the solution, or MNs are partially retracted and then re/inserted to ensure relaxation of the compressed tissue around the MN tips [6].

Solid MNs are usually made from stainless steel [13,47], silicon [67], nickel [68], titanium [69], or polymers [70] so they pierce the SC to allow the drug to pass through to reach the lower layers of the skin where the diffusion rate is faster [5]. Usually, they deliver drugs transdermally by one of three mechanisms, which include “*poke and patch*”, “*poke and release*”, and “*coat and poke*” approaches [71]. The “*poke and patch*” is a mechanism of applying MNs to pierce the SC to create transient aqueous microchannels on the skin surface before the application of a drug-loaded patch or other topical drug formulation, which can be in the form of a gel, ointment, cream, solution, foam, lotion, or spray (Figure 1B) [8,14,58]. Then, a drug from a formulation or patch permeates through these microchannels by passive diffusion directly into the dermis to reach systemic circulation [6,14], improving the drug permeability up to four orders of magnitude [14,67]. Compared to hollow MNs, solid MNs are easier to manufacture and have better mechanical strength [5], but there is no precise control over the drug dosage, and the drug solution should be easily self-applicated [72]. A variation of the “*poke and patch*” approach is the “*scrape and patch*”, where MNs or microblades first scratch the skin to form microabrasions and then a patch is applied with the drug solution [71]. This approach was used by Mikszta et al., where blunt MNs, termed microenhancers, delivered Coomassie blue to the epidermis [73]. In the second case, the “*coat and poke*” mechanism involves the coating of solid MNs with a suitable drug formulation, so that when applied to the skin, the drug continuously dissolves and deposits, after which MNs are withdrawn (Figure 1C). This type of MN is a single-unit drug delivery system, which serves as a puncturing skin device and drug reservoir [37] for a wide range of drugs, including both hydrophilic or hydrophobic drugs with low molecular weight, nucleic acids [43], proteins [74], peptides [75,76], or particles. A major obstacle to this approach is the limited quantities of the drug that can be applied on MN bases and shafts [6,37], as thick coatings, due to the reduced sharpness of MNs, lead to poor drug delivery efficiency into the skin. Coated MNs are, therefore, only suitable for very potent drugs such as vaccines [6,77,78,79].

Dissolving MNs are used to slowly release drugs into the skin by the “*poke and release*” approach (Figure 1D) [14]. They consist of a soluble matrix containing biodegradable material such as a biodegradable polymer or sugar and encapsulated therapeutic agents in their matrix [6,14,71] and they function according to a one-step application principle [14], as biodegradable MNs dissolve after contact with the interstitial fluid, thus releasing the incorporated drugs [6].

Various materials, including poly(vinyl alcohol) (PVA), poly(vinylpyrrolidone) (PVP), carboxymethyl cellulose (CMC), chondroitin sulfate, and sugars such as dextran, galactose, or maltose have been used to produce this MN type [84,85,86,87,88]. As water-soluble materials are usually used for dissolving MN production, the potential of leaving biohazardous, sharp waste is low, allowing safe disposal of the remaining device [6]. Since the drug release kinetics depend on the degree of dissolution of the incorporated polymers, it is possible to adjust the sustained drug delivery by selecting the proper polymer composition or modifying the manufacturing process [6]. However, the main disadvantage is the deposition of polymers in the skin, which is undesirable for long-term use [6].

Hydrogel MNs are the fifth type of MNs, which consist of a hydrogel-forming matrix [89] and can be used for TDD when a drug is incorporated into cross-linked polymer microprotrusions. They swell after application to the skin, absorb interstitial fluid from the tissue, and subsequently induce drug diffusion through the swollen MNs [6,48]. Usually, they are made from aqueous mixtures of polymeric materials such as polymethylvinylether-co-maleic acid (PMVE/MA) [6]. Besides that, hydrogel MNs are suitable for real-time monitoring of analytes in body fluids as they swell after insertion into the skin and subsequently collect interstitial fluid (Figure 1E) [90]. These swollen patches can be analyzed to gather information on the current status of analytes or biomarkers in body fluids, thus enabling painless, continuous monitoring and disease management [48].

Regardless of MN type, their success is highly dependent on their fabrication technique, which should enable reproducible production of MNs, painless application, and efficient drug delivery to the systemic circulation. As mentioned before, traditional manufacturing methods possess many drawbacks and 3D printing has emerged as a new manufacturing approach that can exploit the potential of the TDD through MNs and fabricate these tiny complex geometric structures.

## 3. Fabrication of Microneedles Using 3D Printing Technologies

The process of 3D printing (3DP) of MNs usually contains three main steps. Firstly, a 3D object is designed with computer-aided design (CAD) software, and the geometry is optimized according to printer specifications. Then, the 3D object is exported to a common and printer-recognizable file format such as standard triangulation language (STL), which includes only 3D geometry in the form of each vertex’s position data or an OBJ file in which additional information about polygonal faces or color texture is coded [91]. Finally, the object is printed in a layer-by-layer manner [92].

Different 3DP technologies have been developed and they can be classified according to the energy source, material source, or other mechanical characteristics. In the pharmaceutical field, the most common 3DP technologies are [25,93]:

1. nozzle-based deposition systems (fused deposition modeling, FDM),
2. laser-based writing systems (stereolithography, SLA; digital light projection, DLP; liquid crystal display, LCD; continuous liquid interface production, CLIP; selective laser sintering, SLS; direct metal laser sintering, DMLS; selective laser melting, SLM; two-photon polymerization, 2PP, etc.), and
3. printing-based inkjet systems (continuous inkjet printing, drop-on-demand printing).

A summary of the nozzle-based deposition systems and laser-based writing systems and their advantages and disadvantages are provided in Table 1. The printing-based inkjet systems will not be discussed further, but readers can find additional information in these papers [94,95,96,97].

### 3.1. Nozzle-Based Deposition Systems

#### Fused Deposition Modeling (FDM)

One of the most popular AM techniques is extrusion-based fused filament fabrication (FFF), also referred to as fused deposition modeling (FDM). This user-friendly AM technique was introduced commercially in the early 1990s by Stratasys Inc., Edina, MI, USA [114].

**Figure 2:**
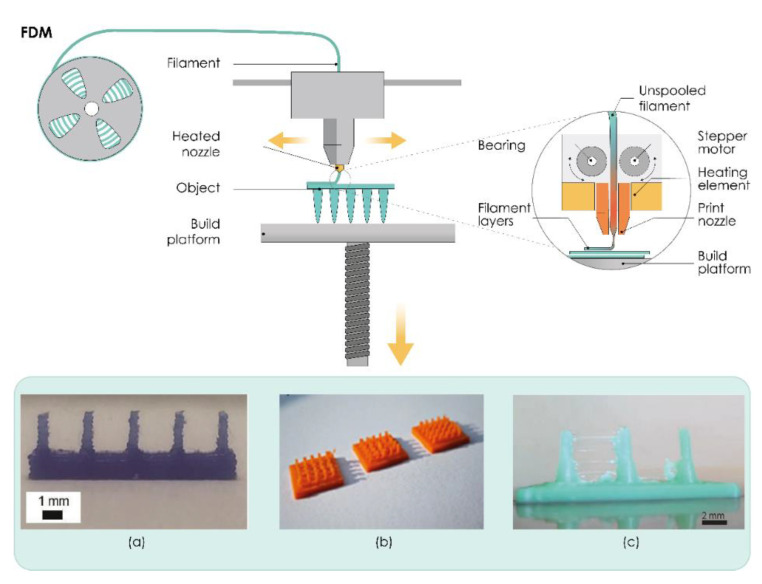
The working principle of fused deposition modeling (FDM). (**a**–**c**) Microneedles produced by FDM technology. Reproduced with permission from [26,32,117], Royal Society of Chemistry, London, UK, 2018 (**a**), Springer Nature, Basingstoke, UK, 2020 (**b**), and John/Wiley & Sons, Inc., Hoboken, NJ, USA, 2020 (**c**). The image was created with Adobe Illustrator CC (Version 23.0.1.; Adobe Inc., San Jose, CA, USA, 2019).

In this process, the suitable thermoplastic material, in the form of a filament, is melted in a liquefier head at a temperature above its melting point and then selectively deposited layer-by-layer through a nozzle on a build plate, where it is cooled and solidified in less than a second, as shown in Figure 2. The printer’s head moves within the *x*- and *y*-axes, whereas the platform can move within the *z*-axis, thus creating 3D structures [98].

The quality and mechanical properties of the fabricated part can be attributed to the proper selection of process parameters such as nozzle diameter, feed rate, the temperature of both the nozzle and the building plate, printing speed, the height of the layers, and orientation of the built part. All these process parameters need to be studied and optimized in the FDM process to improve surface finish, strength, and other properties of the printed part [99,115].

The dimensions of filaments adapted in the commercially available FDM print head are in the range of 1.75 mm and 2.85–3 mm and their essential property is thermoplasticity [91]. Although this technique is affordable, highly reliable, fast, and uses relatively inexpensive materials, FDM suffers from low resolution compared to other AM techniques [28,98] and printed objects face the inherent limitations in dimensional accuracy and surface texture [116].

Initial attempts to use FDM processes in the production of MNs were reported by Luzuriaga et al. (Figure 2a). To obtain ideally sized and shaped MNs that can insert, break off, and deliver drugs into the skin and to overcome printer resolution issues, this research group developed a post-fabrication chemical etching protocol. Different types of MN arrays were printed using polylactic acid (PLA), an FDA-approved, renewable, biodegradable, thermoplastic material, followed by chemical etching using an alkaline solution. Results showed that this post-fabrication etching step does not affect the mechanical and material properties of PLA-fabricated MNs and can be used to obtain sharp MN tips [26]. Camović et al. also successfully employed FDM followed by a chemical etching process to obtain solid PLA MNs with desirable shape and size (Figure 2b) [32,118]. Tang et al. investigated the effects of FDM process parameters, such as printing temperature, layer thickness, extrusion width, infill width, and nozzle orifice diameter on the final print quality of MNs printed with different types of PLA (Figure 2c). It was found that a thinner layer led to a more accurate tip, and a thinner infill width resulted in more accurate part diameters. It was also reported that the smaller nozzle orifice and increased spacing between MNs produced better surface finish but had no significant effect on part accuracy [117].

Derakhshandeh et al. used an FDM printer to produce hollow MNs and integrated them into personalized and programmable bandages with the capability of actively controlling the release profile of multiple drugs, such as vascular endothelial growth factor (VEGF), in the treatment of chronic wounds. The authors believe that this tunable platform can be an efficient alternative for the current wound care methods. The effectiveness of the MNs in transferring the active compounds through the wound crust and necrotic tissues were successfully demonstrated in vitro. In vivo transdermal delivery of VEGF to chronic wounds of diabetic mice by this platform successfully enhanced wound closure, re-epithelialization, angiogenesis, and hair growth [119].

### 3.2. Laser-Based Writing Systems

#### 3.2.1. Stereolithography (SLA)

Photopolymerization-based or photocuring 3DP technologies, which are based on the ability to selectively polymerize photosensitive polymers through laser emissions or projections of light, are one of the oldest and the most widely used AM technologies in the current manufacturing industry [120]. Vat photopolymerization technologies such as stereolithography (SLA) are liquid-based processes characterized by high precision and accuracy. SLA is based on the controlled layer-by-layer solidification of a photosensitive liquid resin when scanned by a laser beam. Laser tracks and “draws” each layer, curing the resin as it travels along the x–y plane [121]. SLA printed parts are considered isotropic, which means there is no risk of delamination [102].

**Figure 3:**
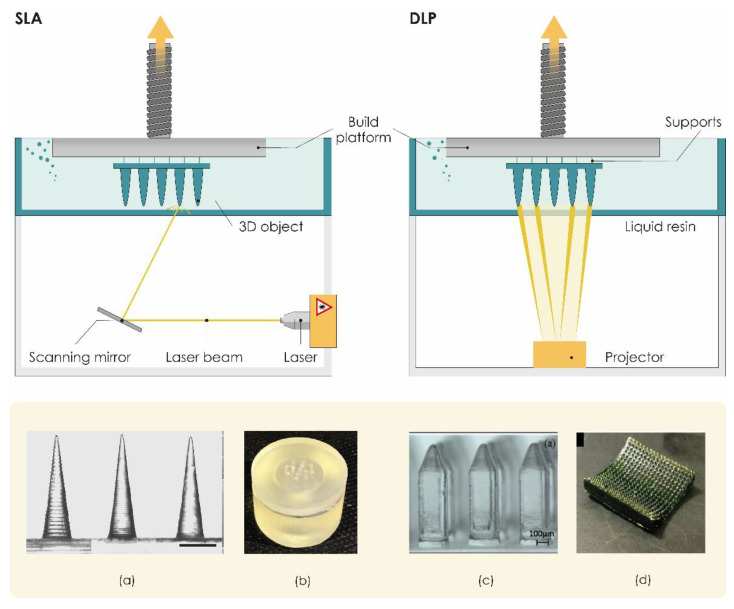
The working principle of stereolithography (SLA) and digital light projection (DLP). (**a**,**b**) Microneedles manufactured by SLA. Reproduced with permission from [122,123], Springer Nature, 2019 and MDPI, 2018. (**c**,**d**) Microneedles manufactured by the DLP technique. Reproduced with permission from [124,125], IOP Publishing, Ltd., Bristol, UK, 2015, 2020 The image was created with Adobe Illustrator CC (Version 23.0.1.; Adobe Inc., San Jose, CA, USA, 2019).

SLA printers usually include a printing platform and a resin tank (Figure 3). A UV laser draws the cross-section onto a photopolymer resin bath that solidifies the cross-section. Once the first layer is completed, the platform is typically lifted about 0.05–0.15 mm according to the layer thickness [103], the laser then solidifies the next cross-section, and the process repeats until the entire part is finished. Resin that is not touched by the laser remains in the vat and can be reused. A post-process treatment may be used to achieve the desired mechanical performance [28]. The SLA processes can be divided into two main categories based on different filling mechanisms: free surface and constrained surface. In the free surface approach, structures are built bottom-up from a support platform that rests just below the resin surface while the constrained surface approach, also called the “bat” configuration, has a building platform which can be suspended above the resin bath [121].

To date, SLA is the most widely reported 3DP technology in the development of transdermal MNs, characterized by low printing costs, and the ability to fabricate solid features smaller than 100 µm. Given all these advantages, we believe that this 3DP technology will open new horizons for the development of TDD.

A study conducted by Pere et al. in 2018 proved that SLA 3DP technology can serve as an effective technology for the manufacturing of solid MN patches with excellent mechanical strength and piercing capacity. MNs with cone and pyramid geometries were printed using a biocompatible Class 1 polymer as a resin and then coated with insulin–sugar films by inkjet printing. The printer resolution enabled the formation of sharp needle tips that facilitate piercing the skin and Franz cell diffusion studies revealed rapid insulin release rates within 30 min [33].

In the research conducted by Economidou et al., pyramidal and flat spear-shaped MN arrays for intradermal insulin administration were also produced with biocompatible resin as printing material. The authors improved the printability of MNs using the printing-in-an-angle approach and reported that the optimization of the printing process resulted in a significantly improved skin penetration ability of SLA 3DP MNs compared to metallic MNs of similar geometry. Results from in vivo trials on diabetic mice showed rapid insulin action with excellent hypoglycemia control and lower glucose levels within 60 min compared to subcutaneous injections [126].

Xenikakis et al. produced and evaluated in vitro SLA 3DP solid MNs for TDD. Results showed that MN arrays made of polymer-based material can withstand extreme forces without breaking and can sufficiently penetrate human skin with an insertion force of 1.1 N, a force that allows the manual application. To accurately simulate the insertion process of the printed MN arrays, finite element analysis (FEA) was used, and the authors believe that this analysis can provide a framework for predicting puncture load. Permeation studies have shown that 3DP MNs can significantly improve the transport of the two model dyes with different molecular weights across human skin [127].

Uddin et al. also successfully employed SLA 3DP to obtain polymeric MN arrays. Printed MNs were then coated with an anticancer compound (cisplatin) using inkjet coating as the most promising coating approach. The unique cross-shaped MN design enabled the coating quantity to be increased from a few micrograms to milligrams compared to previously reported designs due to the increased surface area. Results from penetration studies through porcine skin showed that the maximum penetration force required for the SLA 3DP MNs was significantly lower than the one required for the metallic ones. This study also demonstrated the potential for in vivo transdermal delivery of cisplatin to A-431 epidermoid skin tumors using 3D printed MN patches. Rapid cisplatin release rates of 80–90% within 1 h were revealed using Franz cell diffusion studies and in vivo evaluation using Balb/c nude mice presented sufficient cisplatin permeabilization with high anticancer activity and tumor regression [96].

The application of SLA 3DP enables researchers to produce not only MN arrays but also MN master molds. Conventional methods employed in the production of MN masters, including microelectromechanical system fabrication and micromachining, are usually costly and time-consuming. Krieger et al. developed, a two-step “print and fill” fabrication method using a low-cost desktop SLA 3D printer that allows researchers to produce in-lab MN master molds rapidly without expensive manufacturing facilities or expertise in microfabrication. Using the molds obtained from the “print and fill” method, both carboxymethyl cellulose loaded with rhodamine B as well as polylactic acid MN arrays were produced and their quality examined (Figure 3a) [122].

In addition to the research interest in 3D printing of solid MNs, researchers also introduced the use of 3D printers to fabricate hollow MNs. A custom 3D printed hollow MN biodevice (Figure 3b) consisting of a reservoir chamber and an array of conical MNs was successfully manufactured by Farias et al. using SLA and the proprietary methacrylate-based photoresin as printing material [123].

Yeung et al. introduced an interesting approach for microchannel-MN platform 3D printing. They used SLA 3DP to manufacture a single-piece, multi-inlet, 3D microfluidic device with an embedded hollow MN array. This device, consisting of a hydrodynamic mixing module that allows homogeneous mixing of multiple fluids under different flow rates, is interfaced with a hollow MN array able to transdermally deliver mixed drug solutions and facilitating programmable new TDD applications [64].

Recently, Economidou et al. also demonstrated that SLA 3DP can be a valuable tool for the fast and cost-effective production of hollow MN patches. For the first time, this research group presented a universal TDD device consisting of a hollow MN patch integrated with a microelectromechanical system (MEMS). SLA 3DP hollow MNs were paired with a diaphragmatic micropump as a MEMS, to create a 3D printed MN-mediated drug delivery system that enables personalization of the treatment through in situ control of drug administration by the user. In vivo results showed that administering insulin with this innovative device improved glycemic control in diabetic mice compared to subcutaneous injections [102].

Although SLA technology can be very successfully used to manufacture MN arrays and MN-mediated TDDs, the successful commercialization of such products will require numerous additional investigations regarding optimization of printing and post-printing parameters and selection of appropriate biocompatible and safe printing material.

Economidou et al. recently made an important contribution in this context by investigating the effects of printing angle and post-printing curing conditions on the sharpness and mechanical properties of SLA-printed MNs. It was found that the post-printing curing conditions influenced the mechanical properties of the material and MNs due to the degree of cross-linking. These results suggest that optimizing the post-printing curing regime is a crucial step for the photopolymerization of printed MNs. The printing angle proved to be very important as it also influenced the MN quality and dimensional accuracy, where the print quality at 45° seemed to improve significantly and the MN appeared sharper without any structural manufacturing faults being detected. Results also suggested that MN geometry and geometrical parameters influenced piercing force and coating morphology and dissolution, respectively [128].

#### 3.2.2. Digital Light Processing (DLP)

Digital light processing (DLP) is photopolymerization-based technology that differs from SLA only in the light source used (Figure 3). DLP is usually faster than SLA, as a high-resolution intelligent projector (digital micromirror device, DMD) illuminates the entire cross-section of the object at once in the form of volumetric pixels and the entire layer is produced simultaneously [7]. The technology is characterized by high printing resolution, with a minimum size of 50 μm, and the ability to print objects with a smooth surface. On the other hand, DLP 3D printers are very expensive [92].

Miller et al. created a hollow MN array out of a photosensitive acrylate-based polymer resin using DMD™-based microstereolithography [129]. Gittard et al. also utilized DMD™-based stereolithography to obtain solid MN array structures in various geometries from an acrylate-based polymer. To coat printed MNs with silver and zinc oxide to provide an antimicrobial effect, this research group used the pulsed laser deposition technique [130]. During their experimental work, this group noticed discrepancies between input and measured dimensions of MNs, which was ascribed to translation of the STL model to the physical structure, or factors such as diffraction, refraction, and photoinitiator diffusion.

Boehm et al. combined visible light dynamic mask microstereolithography and indirect rapid prototyping (e.g., micromolding) to prepare MNs from biodegradable acid anhydride copolymer Gantrez^®^ AN 169 BF (Ashland, Wilmington, NC, USA). MNs were then loaded with miconazole using a piezoelectric inkjet printer [131,132].

In their work, Lu et al. developed poly (propylene fumarate) PPF-based MNs arrays (Figure 3c) using multi-material microstereolithography (μSL). Their approach was to incorporate the chemotherapeutic drug dacarbazine into the PPF matrix before the photopolymerization process. However, it was demonstrated that this promising approach to fabricate drug-containing MNs is limited to radiation-immune compounds [124].

A very interesting approach by Lim et al. showed that DLP can also be a suitable technique for the fabrication of MNs on personalized contoured surfaces. They developed a personalized, dual-function MN splint via a DLP 3D printer that can immobilize the affected trigger finger and deliver diclofenac through MN-assisted transdermal drug delivery for pain relief [45]. Using DLP 3DP, Seng Han et al. developed a personalized MN eye patch to create microchannels to improve permeation of antiwrinkle peptides (Figure 3d) [125]. Other research groups utilized DLP 3D printing technology for the fabrication of MN master molds [133,134,135]. El-Sayed et al. successfully used a desktop DLP 3D printer to produce positive master molds. To enhance the insertion of the MNs in the skin, they designed “tanto blade”-inspired dissolving MNs [134].

Yao and his research group introduced hydrogel MNs fabricated by a high-precision digital light processing (H-P DLP) 3D printing system. A self-built high-precision digital light processing (H-P DLP) system based on light-curing [136] was utilized to print MNs of many shapes with biocompatible materials with different printing parameters. Their results showed that the stiffness and precision were significantly influenced by the exposure time of each layer [137].

#### 3.2.3. Liquid Crystal Display (LCD)

**Figure 4:**
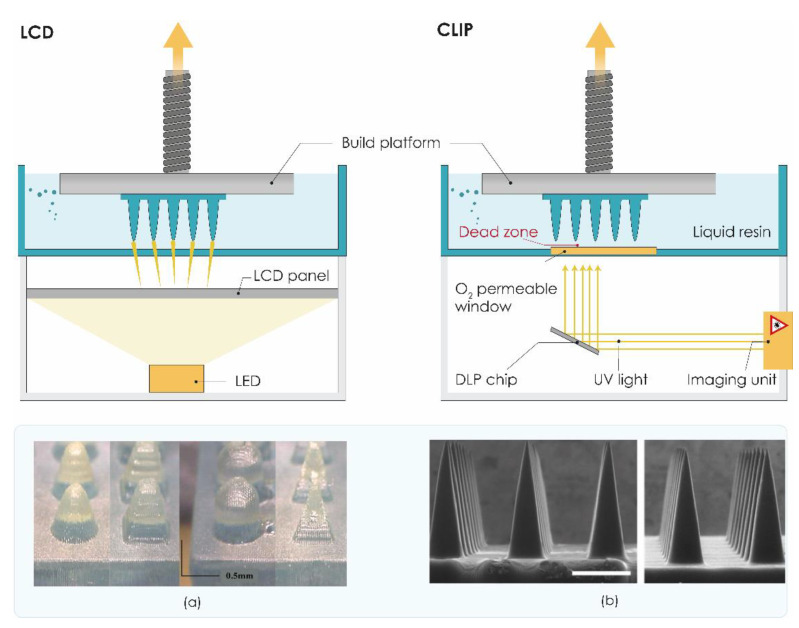
The working principle of liquid crystal display (LCD) and continuous liquid interface production (CLIP). (**a**) Microneedles produced by LCD. Reproduced with permission from [138], Elsevier, 2021 and (**b**) CLIP technology. Reproduced with permission from [139], PLOS, 2016. The image was created with Adobe Illustrator CC (Version 23.0.1.; Adobe Inc., San Jose, CA, USA, 2019).

Another vat polymerization technology, namely liquid crystal display (LCD), is based on UV-mediated resin solidification. In LCD 3DP, the liquid crystal display is used as an imaging system. This bottom-up 3D printing method has several advantages over top-down processing, such as the DLP 3DP system includes a smaller volume of resin required during fabrication and has the capability of achieving a high vertical resolution and a shorter curing time (Figure 4) [104].

Printing accuracy and light intensity are the main differences between DLP and LCD 3D printing, where the intensity of LCD 3D printing is very weak, and the precision of LCD printing technology is inferior to DLP. Nevertheless, LCD 3DP offers satisfactory resolution down to 150 μm in the horizontal direction and 50 μm in the vertical direction, enabling the low-cost production of microstructures with complex architectures [92].

Recently, Xenikakis et al. presented for the first time the fabrication of a hollow MN device consisting of an array and a reservoir (Figure 4a) by the LCD method for transdermal peptide delivery. Hollow MNs were manufactured using LCD from biocompatible resin material, while reservoirs were fabricated using FDM 3DP from PLA filament. Their results indicated that the 3DP hollow MN device possessed proper physical characteristics with qualified mechanical properties and adequate skin penetration ability. Triangular pyramid MNs with a printing angle of −52.63° were established as the most promising geometry for permeation studies. Hollow MNs were also evaluated for their mass flow capability [138].

#### 3.2.4. Continuous Liquid Interface Production (CLIP)

In 2015, Carbon 3D Corp (Redwood, CA, USA) developed new continuous liquid interface production (CLIP) technology as a novel alternative to traditional layer-by-layer SLA [106]. The invention of the oxygen permeation membrane, which helps the consecutive printing for the oxygen permeation to inhibit the radical polymerization, is the key to this technology [92]. The process begins by directing ultraviolet (UV) light through an oxygen-permeable window into a pool of photopolymerizable liquid resin, which is selectively polymerized by UV light. Above the window, a liquid “dead zone” of non-polymerized oxygen-inhibited resin is maintained, enabling continuous rather than layer-by-layer production of the part (Figure 4) [105,106]. Although CLIP technology allows fast production of high-resolution structures, the technology is costly and not readily available or convenient for in-house MN manufacture.

Only one year after its introduction, the CLIP technology was successfully used to rapidly prototype MNs for TDD. Johnson et al. utilized the CLIP technique to rapidly manufacture sharp MNs with different geometries in one step (Figure 4b). These MN patches have been fabricated from a range of biocompatible materials. Results indicated that CLIP MNs exhibit sufficient strength to pierce murine skin ex vivo and also showed successful delivery of a fluorescent drug surrogate (rhodamine) into the skin [139].

Free radical photopolymerization, which occurs during the CLIP process, has the potential to damage encapsulated therapeutics, particularly sensitive biologics. On the other hand, the stability of the API incorporated in dissolvable, degradable, or swellable polymer MNs must be maintained during the MN fabrication process. To avoid possible destabilization of encapsulated cargo during the CLIP process, Caudill et al. proposed to coat therapeutic agents on the surface of CLIP-assembled MNs [140]. In their work, they utilized CLIP technology to fabricate MN arrays as well as coating solution reservoirs, called coating mask devices, to coat polyethylene glycol-based CLIP MNs with model protein cargoes, including bovine serum albumin, ovalbumin, and lysozyme, in a spatially controlled manner. MNs were found to rapidly release their coated cargo both in solution and in porcine skin. These protein-coated CLIP MNs were also applied in vivo and showed sustained retention of protein cargo in the mice’s skin over 72 h [140].

#### 3.2.5. Two-Photon Polymerization (TPP/2PP)

**Figure 5:**
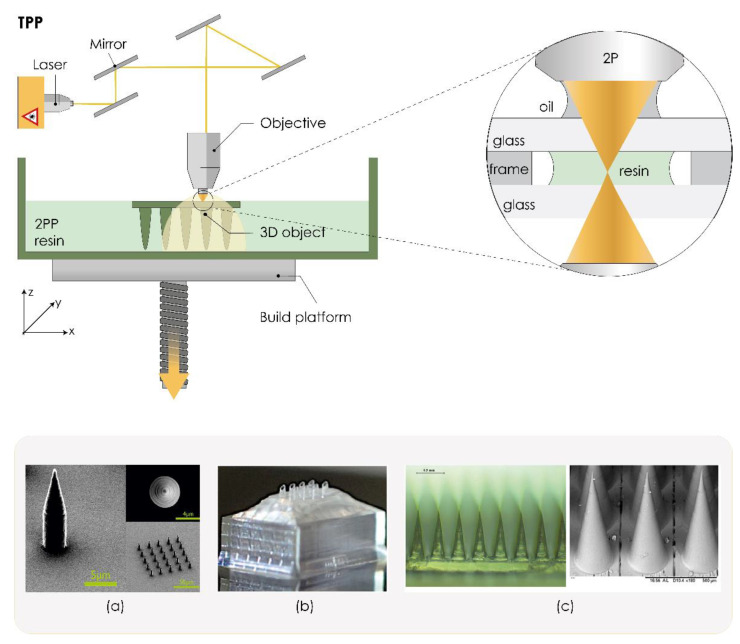
The working principle of two-photon polymerization (TPP) technology. (**a**–**c**) Microneedles produced by TPP. Reproduced with permission from [17,142,143], IOP Publishing, Ltd., 2020, John Wiley & Sons, Inc, 2017, Springer Nature, 2020. The image was created with Adobe Illustrator CC (Version 23.0.1.; Adobe Inc., San Jose, CA, USA, 2019).

As the most accurate 3DP technology to date, the two-photon polymerization (TPP) or 2PP technique enables layer-by-layer fabrication of 3D structures from solid, liquid, or powder precursors for microscale and nanoscale structures. Induced by a near-infrared femtosecond laser, TPP can fabricate arbitrary and ultraprecise 3D microstructures with high resolution (100 nm lateral resolution and a 300 nm axial resolution) [107]. The technology is based on two-photon absorption. Briefly, a drop of resin is placed on a glass substrate, which is followed by focusing the laser beam of an ultrafast (for example, femtosecond) laser directly on a photosensitive material, so the polymerization process is initiated by two-photon absorption within the focal region (Figure 5) [141].

This fast and one-step process offers several advantages over conventional manufacturing techniques for scalable mass production of MNs. TPP can easily be scaled up for industrial use, there is no need for cleanroom facilities, and it can use various low-cost materials such as ceramics, polymers, and other photosensitive materials. Many research groups have exploited this technique to fabricate solid [108,109,110] or hollow MN arrays [143,144,145] as well as reusable MN array molds [17,146].

Doraiswamy et al. were the first to report using TPP to produce MNs from non-toxic and biologically inert Ormocer^®^ (organically modified ceramic, Fraunhofer-Gesellschaft, Munich, Germany). Obtained MNs exhibited appropriate mechanical properties, and specific penetration without fracture, against porcine skin surfaces [147]. Ovsianikov et al. suggest that TPP can create in-plane and out-of-plane hollow Ormocer^®^ MNs with a larger range of geometries than conventional microfabrication techniques [61]. Gittard et al. suggested that TPP can produce MNs with a wide range of geometries [108]. Another research group printed cylindrical, conical, and pyramidal MNs with excellent biocompatibility by TPP technology and then endowed them with magnetic properties through coating with iron (Figure 5a) [142].

Hollow MNs combined with internal laser-generated microchannels were developed by Trautmann et al. using TPP technology. This novel hybrid approach combining TPP-printed MNs with femtosecond laser-generated microfluidic channels provides an important step towards versatile medical point-of-care systems [144]. Moussi et al. also demonstrated a single fabrication process using TPP 3DP that allows for producing hollow MNs directly connected to a reservoir, making them suitable for implanting inside the body, transdermal sampling, or drug delivery applications (Figure 5b) [143]. Szeto et al. demonstrated that TPP 3D printed hollow MNs can facilitate the intracochlear sampling of perilymph [148]. Cordeiro et al. described an approach to fabricate high-quality MN array master templates using TPP 3DP. These reusable MN array molds were then used to produce dissolving and hydrogel-forming MN arrays (Figure 5c) [17]. Recently, Balmert et al. described a comprehensive approach to produce dissolving MNs with undercut MNs incorporating multiple cargoes for effective multicomponent cutaneous vaccination [149].

In 2019, two-photon grayscale lithography (2GL^®^) was introduced as an innovative high-precision AM technology that combines the extraordinary performance of grayscale lithography with the precision and flexibility of TPP technology. This interesting technology is very accurate and ultra-fast, offering very high spatial resolution in the submicron range. Indeed, the minimum lateral size of the printed voxel can be as small as a few hundred nanometers. We believe that this maskless lithography technique will contribute to a new era of high-precision manufacturing of MN arrays [150].

#### 3.2.6. Powder Bed Technologies (SLS, DLMS, SLM)

Powders can also be used as materials for 3D printing by applying a high-energy beam in technologies such as selective laser sintering (SLS), selective laser melting (SLM), and direct metal laser sintering (DLMS). These techniques require a high energy flow on the powder bed of building material to produce the desired objects [151], while the solidification process of powders can be achieved by sintering or melting [91].

**Figure 6:**
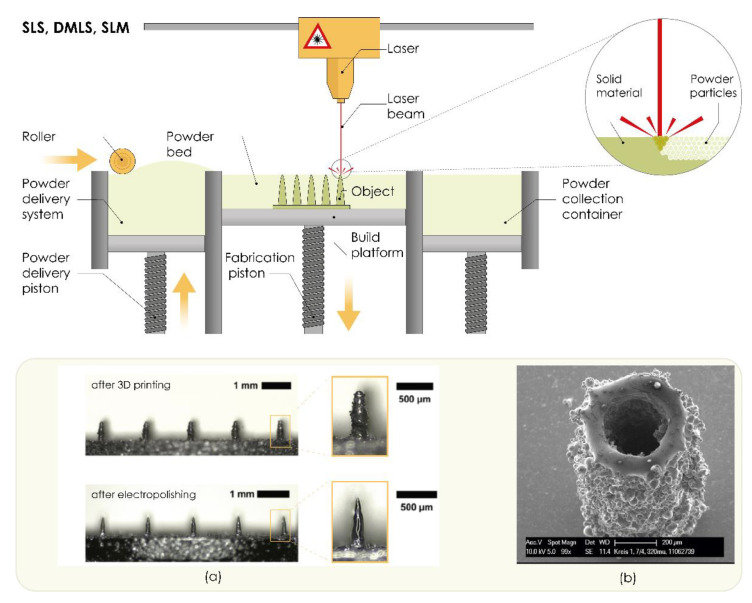
The working principle of powder bed technologies (selective laser sintering, SLS; direct metal laser sintering, DMLS; and selective laser melting, SLM). (**a**) Fabrication of microneedle arrays using DMLS and post-processing by electropolishing. Reproduced with permission from [156], John Wiley & Sons—Books, 2020. (**b**) Fabrication of hollow microneedle using SLM. Reproduced with permission from [158], Walter de Gruyter and Company, Berlin, Germany, 2012. The image was created with Adobe Illustrator CC (Version 23.0.1.; Adobe Inc., San Jose, CA, USA, 2019).

Selective laser sintering (SLS) is powder bed 3D printing technology invented by Carl R. Deckard for his master’s thesis at the University of Texas and was patented in 1989 (US 4863538 patent) [152,153]. This technology produces 3D objects layer-by-layer [154] using a computer-controlled, high-power laser that selectively heats and fuses tightly compact, small powder particles such as metal, plastics, polymers, or ceramics. These particles then solidify and form a 3D structure with the desired shape and properties [91,100,151,155]. Direct metal laser sintering (DMLS) is an extension of the SLS process and produces 3D printed objects by sintering the powdered metals with a precise, high-intensity laser [154]. The SLS and DLMS systems consist of a laser system, powder bed, and spreading platform [155]. In DLMS, the powder bed is filled with metal alloy powders such as bronze, steel, stainless steel 316 L, titanium, or Al-30% Si without a binder or fluxing agent [112]. The 3D process starts with CAD data exported in the industry-standard exchange file format, STL [153]. Subsequently, the powder is distributed uniformly onto the building platform by using a slot feeder and a scraper blade (roller) that even the surface [28,155]. Sintering means that the printing process is maintained at a temperature that does not completely melt the powders, as it is below the melting point of the material, which is sufficient to enable fusion between particles at the molecular level [28,91,153,155]. The powder bed is lowered by the thickness of one layer so that a subsequent layer of powder is loaded into the build tank and fused by the laser. The process is repeated until the final 3D model is created (Figure 6) [100,155]. Support during the 3D printing process is provided by unsintered powder particles present on the build platform, which are then removed by hand, a vacuum, or sieving [28,91,155].

DMLS can be used to produce small, geometrically complex, fully functional metal parts such as MNs from medical-grade materials with great freedom of design, which would be difficult to produce with classical methods [112,156]. The main advantages of these methods are the production of objects with satisfactory accuracy, resolution, and mechanical properties of the finished parts [112] and without residual stresses and internal defects that can affect conventionally manufactured metal parts [113].

Sun et al. [157] investigated the potential of three different 3DP techniques for printing MN-like structures: DMLS of stainless steel (SS) 316 L, the lost-wax casting of sterling silver using DLP/SLA-printed wax masters, and binder inkjet printing of SS 316 L. Results showed that binder inkjet printing of SS 316 L MNs was associated with the smallest in-plane offset, out-of-plane offset, and eccentricity of nominally symmetric features. The minimum feature size was approximately 285 μm and the cylinders had straight sidewalls and fairly flat top surfaces. Furthermore, this technique enabled its independent optimization to manufacture final parts with high dimensional accuracy. In contrast, DMLS produced smaller MNs (185 μm) because of a significantly larger offset, but the edges of printed parts were less sharply defined than the samples produced with binder inkjet printing [157].

Krieger et al. [156] used a DMLS 3D printer to produce solid MNs from a medical-grade 316 L stainless steel powder. Although MN dimensions in the specified design input file were 1000 μm in height and 250 μm in base diameter, it was noticeable that printed MNs were significantly shorter (657 ± 16 μm). Since printed MNs were not sharp and had a rough surface, the MNs were post-processed by electropolishing (Figure 6a). This process did not affect the height of printed MNs, while electropolished MNs had a smooth surface and satisfactory tip sharpness, as the tip radius was reduced by 62% compared to unpolished MNs (from 51 μm to 19 μm). These MNs were further used as an alternative to commercial wet Ag/AgCl electrodes for EMG signal acquisition. The human study involved 14 healthy volunteers, who showed good tolerance of MN electrodes for up to 6 h. Future studies are, however, necessary to ensure the safe handling and disposal of sharp MNs, with regard to safety concerns such as biocompatibility of the materials and risk of skin irritation [156].

The working principle of SLM is similar to SLS and DMLS in that the powder is spread by a roller on the building platform and a laser fuses the powder on points defined by CAD design data. Subsequently, the platform is lowered and another layer of powder is applied [100]. In contrast, the successive layers of metal powders are fully melted and fused after laser scanning, resulting in superior mechanical properties [28,100]. SLM printers require support structures to anchor the part to the build platform and enable heat transfer away from the print to reduce thermal stress [159].

To produce hollow MNs from stainless steel 316 L, Gieske et al. [158] modified the SLM process and reported a selective laser micromelting (SLμM) setup. They produced MNs with a minimum wall thickness below 50 μm and an aspect ratio of 30:1 (Figure 6b). Produced hollow MNs had a height of 1200 μm, while the minimum average inner diameter was 160 μm [158].

## 4. Parameters Affecting the Mechanical Properties of 3D Printed Microneedles

In order to be considered a successful drug delivery system, the MN array patch must meet several criteria, such as [160,161]:

- insertion into the skin at sufficient depth without breaking,
- dimension and design with optimal properties, and
- using proper (biocompatible) material.

Since various methods of 3D printing of MNs have been developed, it is necessary to carry out optimization studies on the design and dimensions of MNs, but considering the 3D printing quality, which strongly depends on the set printing parameters [128]. However, to the best of our knowledge, no clinical studies have been done and no marketed products of 3DP MNs are available. Further development of 3DP MNs is surely dependent on the flow of other AM studies and on the status of conventional MNs on the market.

### 4.1. Material Selection

It is well known that materials used for MN manufacturing must have sufficient strength to penetrate biological barriers, must be easily manufactured, and be compatible with drug molecules. Material properties affect critical parameters such as stability, tensile strength, drug loading, and biocompatibility of MNs [162,163]. Biocompatibility and safety are of the utmost importance when it comes to material selection for MN manufacturing. Although the AM technology allows the use of different materials (Table 1), all materials must fulfill properties such as biocompatibility, biodegradability without toxic products, mechanical strength, and scalability to be used in the development of TDD systems such as MNs [164]. This can seriously limit the expansion of 3D printing in MN manufacturing, as the material selection can be challenging.

All photocuring 3DP techniques require the printed materials to have a photosensitive property and to be composed of precursors, photoinitiators, adsorbers, fillers, and additives [121]. Most of these materials lack biocompatibility and can therefore be toxic for living cell components [165,166], thus limiting their use in the development of TDD. The exact composition of the commercial resins is usually proprietary and minimal information on the constituents is available, which limits toxicity evaluation.

Methacrylate-based resins are the most commonly used materials in SLA manufacturing of MNs [96,123,126,127]. Acrylate-based resins [125,129,130] as well as biocompatible poly-propylene fumarate (PPF) [124] or polyethylene glycol diacrylate (PEGDA) [137] were used in DLP MN manufacturing. Biocompatible and FDA-approved materials were used in CLIP 3DP MN development, including polyethylene glycol dimethacrylate (PEG) and polycaprolactone trimethacrylate with TPO (diphenyl (2,4,6-trimethyl-benzoyl-)phosphine oxide) [139,140]. Organically modified ceramic (Ormocer^®^) [61,147] or acrylic are commonly used in TPP MN fabrication [109,148].

Mansor et al. investigated several commonly used polymers as candidates for the SLA process. They prepared solid MNs using PVA, PLA, polyester resin, and ABS and characterized their mechanical properties. Results showed that PVA had had the highest ability to withstand force over other applied materials [167].

Standard materials used in FDM printers are thermoplastic filaments such as acrylonitrile butadiene styrene (ABS), PLA, PVA, high-impact polystyrene (HIPS), polyethylene terephthalate glycol-modified (PET-G), and nylon. Biocompatible PLA was predominantly used in FDM MN fabrication [26]. While SLS can be used for a variety of polymers, metals, and alloy powders, selective laser melting (SLM) and DMLS can only be used for certain metals such as steel, stainless steel, cobalt, titanium, and aluminum [28,100].

The choice of materials should be carefully considered to enable safe and effective MN devices to be delivered to patients [147]. Improvements in resins compositions, as well as in-depth studies evaluating the biocompatibility and mechanical properties of the materials used, are mandatory to successfully commercialize MN-based TDD systems in the future.

### 4.2. Precision of 3D Printing

In order to achieve the appropriate product quality, a detailed analysis of the impact of all important process parameters is required, regardless of the 3D printing method. Optimization of process parameters is a key criterion for achieving the appropriate product quality in terms of improving dimensional and geometrical accuracy and surface quality [168].

For MN production, printer resolution is one of the most important parameters for achieving the desired MN design and forming sharp MN tips. Print resolution is influenced by the software and hardware components of the device. Of the software components, the greatest influence on the quality of the product itself is the mesh density when forming the .stl format [169,170,171]. The mesh density directly affects the print accuracy, so the printing of a model with a fine (denser) mesh is more accurate than the printing of a model with a sparse and uneven mesh. The influence of hardware components is reflected in the quality of manufacturing components of the movement and positioning system (number of increments of stepper motors, quality of production of guides, threaded spindles, belts, etc.), extrusion components, i.e., nozzles, heaters (for extrusion-based techniques), and laser system components (for laser-based techniques).

**Figure 7:**
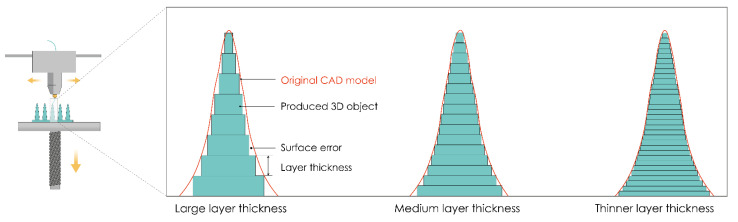
The staircase phenomenon for different layer thicknesses. The image was created with Adobe Illustrator CC (Version 23.0.1.; Adobe Inc., San Jose, CA, USA, 2019).

Layer thickness is one of the process parameters that has the greatest influence on the printing resolution in the z-axis direction (build direction). This influence is especially significant in the building of curved surfaces due to the pronounced “staircase” phenomenon. As the layer thickness increases, the accuracy of the overall finished sample geometry decreases, and the “staircase” phenomenon is more pronounced (coarse surface/edge, Figure 7).

Experimental studies have shown that layer thickness has the greatest influence on the appearance and dimension deviations. It is a parameter that also significantly affects the production time and surface roughness of the finished product [172]. The layer thickness ranges from 10 μm for the SLA process to 400 μm for the FDM process. Therefore, the best results in terms of dimensional accuracy and surface roughness can be achieved by SLA because it allows the printing of samples of a resolution up to 10 µm in the z-direction.

### 4.3. Microneedle Design

Regardless of the manufacturing technique, some of the most important characteristics of MNs, such as insertion and penetration behavior, ability to pierce the skin, or the rate of drug delivery, strongly depend on the geometry of MNs, e.g., MN length, base and tip diameter, shape, and interspace (center-to-center spacing) [19,173].

The length of MNs can usually vary between 150 and 2000 μm and guarantees a direct penetration of MNs through the epidermis layer to the capillary system of the skin in dermal tissue [6]. The length of MNs should be carefully optimized because if the MNs are too long or fragile due to weak materials, the insertion forces can exceed the ultimate tensile forces and cause breaking of the MNs [14,128]. Due to their ability to extract skin interstitial fluid and to monitor numerous biological markers or exogenous molecules, MNs have also been introduced as non-invasive devices for patient monitoring and diagnostics [14,19,48]. For that purpose, the MN length must be at least 900 μm [61].

The radius of the curvature is crucial for efficient MN insertion. If the MNs are blunt or too short, the skin may fold around them during application and prevent their penetration [14,128]. An MN radius of about 50–250 μm at the base and 1–25 μm at the tip ensures that nociceptors, located in the dermis, are not reached and additionally means that MNs remain intact after application [6,174]. The sharpness of the MN tip is also one of the most important factors for penetrating the skin. The sharper the MN tips, the higher the probability for sufficient and effective skin penetration. Larger tip diameters require higher insertion forces [14,128]. However, to ensure the optimal tip radius, it is necessary to use a robust material, as polymer MNs with the same dimensions fracture easily [175]. 3D printing technologies are limited by the resolution of the printer, which directly affects the tip radius (described in Section 4.2).

The significance of MN tip geometry for MN insertion was studied by Davis et al. They measured the force required to insert MNs into living skin and the force that MNs can withstand before fracturing. The insertion force varied linearly with the interfacial area of the MN tip. Increased wall thickness, wall angle, and tip radius resulted in a higher force required for MN fracture. Reduced MN tip radius from 80 to 30 µm improved insertion efficacy because the smaller tip required the lower insertion force to penetrate through the SC [176]. A low tip angle (15–30°) and thin needle shaft (of 120 µm) can effectively enhance MN insertion without causing tensile failure [177]. Comparison of MNs with different bevel angles and the same wall thickness showed that the MNs with lower bevel angle required the least force to pierce the skin and their mechanical strength remained the same, which was confirmed by measuring the force required for their fracturing [178].

The density of the MN arrays is also important for efficient skin penetration [14,128]. It is desirable to avoid a “bed of nails effect”, which occurs when the density of MNs in an array is high. MNs that are close to each other affect each other and the force applied by the inner MNs of the arrays is exerted on an already stretched material [179].

**Figure 8:**
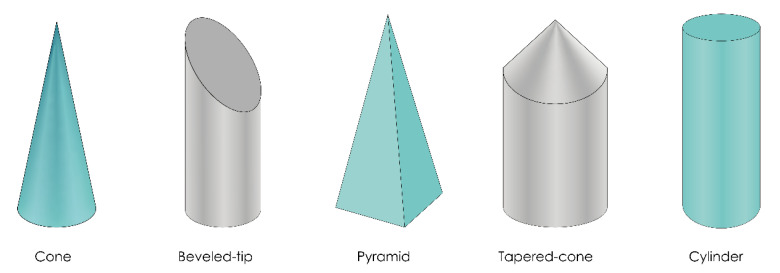
Different shapes of microneedles obtained by 3D printing technologies. The image was created with Adobe Illustrator CC (Version 23.0.1.; Adobe Inc., San Jose, CA, USA, 2019).

The 3D printed MNs may also vary depending on their tip or overall shape, as shown in Figure 8 (e.g., cone, pyramid, cylinder). However, not all 3D printing technologies have the ability to produce all these MN shapes. For example, Luzuriaga et al. and Camović et al. reported that it is quite difficult to obtain a sharp tip of MNs using the FDM method [26,32]. Luzuriaga et al. employed FDM technology to produce MNs with seven different types of shape. Most of them were unable to print because the sharp features exceeded the resolution of the print nozzle, resulting in poor replication of these designs by the 3D printer. Some of the shapes could not be printed due to poor adhesion between extruded layers. As they did not achieve an optimum tip diameter, post-processing in alkaline solution was performed [26]. However, Tang et al. successfully produced a tapered coned MN by the FDM method [117].

The laser-based writing systems are capable of printing MN arrays of the same design with high degrees of consistency [21]. Therefore, MNs with various shapes have been produced by SLA [33,45,64,96,125,126], DLP [124,130,137], LCD [138], CLIP [139,140], TPP [109,142,143,144,148], DLMS [156], and SLM methods [158] (Table 2).

The shape of MNs influences the force required for successful penetration into the skin. Pere et al. concluded that the conical MNs require the least force to penetrate porcine skin compared to pyramid geometries, probably due to the difference in the MN-to-skin contact surface between these two designs [33]. Economidou et al. produced MNs by SLA technology with cone, pyramid, and spear geometries. They found that printing angle has the most crucial influence on the sharpness of MNs, but all designs were found to be mechanically safe for application [128].

Yeung et al. also employed SLA to produce hollow MNs with conical, pyramidal, and fine-tip syringe-shaped designs. They note that pyramidal MNs only left marks on the first layer of parafilm, while the conical ones merely indented the first layer of parafilm. However, the best results were obtained with the syringe-shaped MNs, which left significant imprints on the second layer [64].

It is noteworthy that for optimal results and obtaining MNs with suitable quality and performance, in addition to the manufacturing parameters of 3D printing, the geometric characteristics of MNs also need to be optimized. Manufacturing parameters are notoriously sensitive to 3D printing efficiency, and an application as challenging as MNs necessitates a deep understanding of the relationships between parameters and quality features [128]. Table 2 summarizes the recently reported 3D printed MNs for transdermal drug delivery with detailed geometric features.

## 5. Evaluation of 3D printed MNs

### 5.1. Physical Characterization

Geometry, dimensions, surface morphology, and distribution of MNs on the array can be determined and evaluated by visual inspection, stereomicroscopy, and optical or scanning electron microscopy [180]. Properties of the surface of MN patches can be evaluated by drop shape analysis and contact angle determination [138]. Drop shape analysis implies measuring the contact angles of a liquid drop on a solid surface and capturing a digital image. The image is then analyzed to extract the coordinates for the drop profile and determine the position of the solid–liquid interface. To extract the contact angle from the data obtained, the drop profile is fitted into an equation that is evaluated at the triple-line [181]. The characterization of the MN geometry and the radius of curvature provides important information on the reproducibility of the manufacturing method and provides an opportunity for its improvement to achieve optimum tip sharpness and uniform geometry [180].

Fluorescent labeling or dyeing the molecules incorporated in the MN patch can be used for their identification. Successfully incorporated molecules may be visualized by confocal laser scanning microscopy (CLSM), fluorescent microscopy, or even visual inspection. Visualization is useful for the localization of molecules incorporated within an MN patch whether it is the tip, shaft, or the backing layer [182]. Coated MNs can be evaluated by FTIR spectroscopy [138].

### 5.2. Mechanical Characterization

MNs are exposed to a variety of stresses during insertion due to non-uniformity of the skin surface, unavoidable movements, and stresses exerted upon removal [183]. Mechanical characterization is necessary to ensure the safe use of MNs. MNs tend to bend, fracture, or buckle due to inelastic or elastic instability during insertion or removal, so it is of great importance to evaluate their mechanical properties. Adequate mechanical strength is required to penetrate the SC and deliver the drug. The term mechanical characterization comprises a range of tests that provide the simulation of MNs’ insertion in vivo [6].

#### 5.2.1. Failure Force Tests

The significance of these tests is to determine whether the MNs have sufficient mechanical strength to withstand deformations and any other undesirable changes during handling and skin insertion.

##### Axial Fracture Force Tests

**Figure 9:**
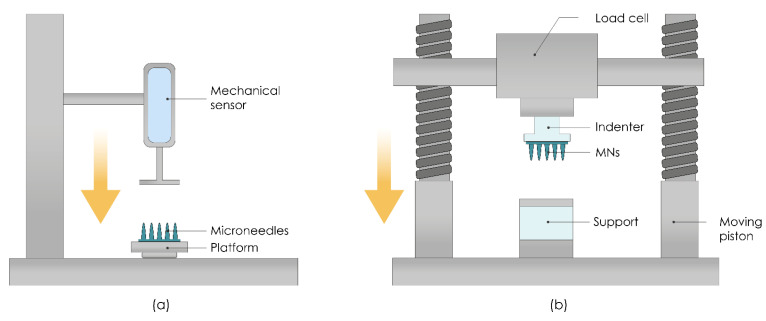
A schematic representation of (**a**) texture analyzer set up for the determination of fracture forces of MN arrays. Reproduced with permission from [186], Elsevier, 2017. (**b**) microneedle penetration testing. Reproduced with permission from [187], Elsevier, 2012. The image was created with Adobe Illustrator CC (Version 23.0.1.; Adobe Inc., San Jose, CA, USA, 2019).

This type of test involves measuring the failure of MNs caused by axial or transverse loading. The test station presses the MN array parallel against a rigid metal surface followed by the measurement of force and displacement while generating the stress against strain curves (Figure 9a). If MNs fail, the force drops suddenly and the maximum force applied immediately before the drop is the force of the MNs’ failure. Subsequently, MN arrays are visually observed by microscope and compared with scans taken before the failure to determine the failure mode [184]. Axial fracture force tests that use only a single MN should be cautiously interpreted because those results cannot always be correlated with ones taken from an MN array [176]. Even individual needles within the same array may fail under different mechanisms due to microstructural heterogeneity within the same MN patch [185]. Another issue to keep in mind is the inaccuracy of the force exerted on the MNs during the compression studies and its difference compared to insertion into the skin. In compression studies, MNs are pressed against a hard metallic surface, and the entire exerted force is concentrated on the contact surface of the MN tip. On the other hand, the forces with which the MNs are inserted into the skin are distributed over a larger MN area, especially following initial penetration, as the flexible skin wraps around the MNs [175].

##### Transverse Fracture Force

Transverse fracture force tests are important for the assessment of the application of MNs, and they can be applied to a single MN, and a row of MNs as well, for which the force should be divided by the number of MNs in a row to calculate the transverse fracture force per individual MN. The main limitation of this test is the required manual alignment of the metal probe with a defined length, which can cause inaccuracies [188]. Failure force under a transverse load can be measured by setting a row of MNs vertically on a metal plate and preparing a glass slide by bonding a polydimethylsiloxane (PDMS) film with cyanoacrylate adhesive to form a stepped structure. This PDMS extension is pressed normally to the axis, starting at the needle tip, using the force–displacement–force test station. Transverse force and displacement are measured until the MNs fracture [184].

##### Baseplate Strength and Flexibility Tests

The fracture of the baseplate during application by the patient is not acceptable, so its strength should also be determined. The flexibility of the MN baseplate ensures appropriate insertion on the skin surface. The degree of flexibility should be sufficient to conform to the irregular topography of the skin without fracturing. To do this, a bending test with three points can be used. Baseplates are placed between two aluminum blocks and the force is applied by a metal probe. The force required to break the baseplate can be measured by observing a maximum peak value on the force–distance curve. The flexibility of the baseplate can be calculated from its bending upon fracture [188].

#### 5.2.2. Insertion Force Tests

Knowledge of the insertion capability of MNs enables the prediction of the appropriate MN length required for their insertion into the skin due to the inherent viscoelasticity of the skin. Measuring the insertion force required to pierce the skin by MNs is also important to ensure a complete and equivalent assessment of fracture forces. Fracture forces should be significantly higher than the insertion forces required for inserting MNs into the skin. MNs insertion can be done manually or by using different applicators (Figure 9b). Applicators offer controlled insertion conditions and lower variability compared to manual insertion. Manual insertion means a wider range of applied insertion forces [6,176]. The required insertion force is about 0.098 N/needle to penetrate the SC [189], but some studies show that an insertion force of only 0.03 N/needle can be sufficient [190].

Due to the inherent viscoelastic properties of the skin, it is difficult to achieve complete insertion of the whole MN length. The depth of MN insertion can be divided into two categories: true depth and estimated depth. Methods that provide true depth include confocal microscopy, X-ray transmission computational tomography (XRTCT), and optical coherence tomography (OCT). The estimated depth of MN insertion can be evaluated by histological cross-sectioning and staining of the skin by colored dyes after MN application. The most commonly used dyes are gentian violet, methylene blue, and trypan blue. These dyes enable the visualization of microchannels generated by MNs in the epidermis. Only the cells of the viable epidermis are colored, while the cells in the SC remain as they were before the MN application. Furthermore, the penetration success ratio of MNs may be calculated as a relation between the number of dye-stained spots observed and the total number of MNs per array [180]. The limitation of dyeing as a method for visual confirmation and measurement of penetration depth of MNs is that lateral diffusion of dyes can lead to overestimation of the micropore diameter. Even if SC is not actually pierced, the dye can accumulate in the indentations and produce false-positive results. A possible alternative might be the injection of the dye into the skin via hollow MNs [183].

Histological cryosectioning implies removing the skin treated with MNs from the bulk skin sample, fixing it in a suitable medium, and then immediately freezing it by using liquid nitrogen and storing it at −80 °C. The thickness of cryosections is between 6 and 12 µm and they are usually stained with hematoxylin and eosin to observe the microchannels created by MNs [191]. This invasive method can affect the dimensions of microchannels produced, as the hydration status of the skin and the tension in elastic tissue change after cryosectioning, which later leads to an overestimation of the measurements [192].

CLSM is an adequate and non-invasive method for measuring the dimension of pores created by inserted MNs. It is based on the exposure of the treated skin area to a solution with specific fluorescent microparticles that migrate down the channels created by MNs. Afterward, these fluorescence probes are detected by CLSM and they indicate the depth of the pores. The penetration depth of CLSM is only 200–250 µm from the skin surface, which limits the possibility of using this method for measuring microchannels made by longer MNs [193,194]. Another disadvantage of this method is the required degree of transparency of the MNs. If the MNs are opaque, such as silicon, metal, or even some colored polymer MNs, they must be removed before imaging, which leads to pore shrinkage and thus to underestimation of the MNs’ pore dimensions [183].

OCT is a good method for evaluating the effects of MNs’ geometry on skin penetration and in-skin dissolution. This non-invasive method allows the determination of the actual depth of MN insertion into the skin in real time. There is no skin excision or mechanical manipulation of samples. It can visualize depths of ~2000 µm [195,196]. It overcomes the problems of the incomparable results of permeability studies conducted on animal skin with those on human skin [197], as well as different skin tension levels caused by transverse mechanical stress in subcutaneous layers in ex vivo human skin models [198]. OCT can provide useful information on skin resealing kinetics following MN removal and in situ dissolution of soluble polymeric MNs, provided that MNs are transparent [199].

XRTCT is another non-invasive method that uses a series of X-ray scans taken at different rotation angles that generate 3D volumetric data, which allows 3D visualization of the MNs’ insertion. It provides the ability to see if all MNs in the array were able to breach and penetrate the skin or if there were regions within the patch that could not penetrate the skin due to mechanical failure. The main limitation of this method is that only MNs made of material with X-ray contrast properties, such as gold, can be observed. It also does not sufficiently distinguish the exact skin layers penetrated by MNs [180,200]. To avoid the use of biological tissue, Larraneta et al. [191] created an artificial membrane made of Parafilm M^®^ (BRAND GMBH, Wertheim, Germany), a blend of hydrocarbon wax and polyolefin, which can be used as a skin model to study the insertion properties of MNs. Parafilm M^®^ simulates the elasticity and mechanical properties of the skin and allows a quick and rapid assessment of the insertion depth of different MNs.

Insertion efficacy can also be evaluated by measuring the transepidermal water loss (TEWL) or the electrical impedance (EI). Both methods are based on the reduction of the barrier function of the SC but cannot provide quantitative information about the insertion depth of MNs [201,202]. Measurement of TEWL is a non-invasive, sensitive method that provides information on the effects of the application of MNs on the integrity of the skin barrier and can be performed in vivo and in vitro. Low TEWL is specific to intact skin, while the application of MNs disturbs the barrier function of the skin and causes increased TEWL. The level of TEWL goes down by the time the skin goes back to its initial condition. The kinetics of pore closure vary due to the treated skin model and type of study (ex vivo or in vivo) [201]. The results obtained by measuring TEWL should be interpreted cautiously as there are many variations due to the differences in permeability and recovery of different skin samples, the lack of universal calibration of the equipment, variability between experimental protocols, differences in temperatures, and the susceptibility to inaccuracies at high vapor flux rates. Data on the depth of MN insertion cannot be obtained from TEWL studies as they are highly sensitive to the skin’s hydration status. In some cases, small changes in water loss cannot be detected if the skin is occluded for several days [81,203]. Measurement of EI is based on a strong inverse correlation between the permeability of the skin and its EI. EI is the phase-dependent resistance of the skin to the flow of alternating current. This method uses the electrical insulation properties of the SC to provide information about skin barrier function and to confirm whether MNs have successfully compromised it [204].

#### 5.2.3. Skin Irritation and Recovery Studies

Depending on the size, material, and the type of the delivered drug, mild and transient erythema may occur as a side effect after MN application. The irritation can be observed dermatoscopically or stereomicroscopically. The degree of skin irritation, although subjective and variable, can be determined by the Draize method in which the skin is observed macroscopically before and after the application of MNs and dermatological changes are evaluated according to the degree of erythema and edema [205]. Skin recovery can also be assessed by monitoring changes in the skin over time using digital photographs [19].

### 5.3. Permeation Studies

#### 5.3.1. In Vitro Permeation Studies

The amount of the drug delivered into the skin can be determined by diffusion cells. Usually, the test is performed on ex vivo human or animal skin or an artificial polymeric membrane placed between receptor and donor compartments. The observed permeant is inserted into the donor compartment, which diffuses into the receptor compartment via the membrane of choice [206]. Transdermal delivery of investigated permeant from MNs can be analyzed from the receptor compartment by high-performance liquid chromatography (HPLC). Intradermal delivery can be examined by extraction and analysis of the compound of interest from the skin sample after permeation [180].

There are two types of diffusion cells: static (Franz type) and flow-through (Bronaugh type). Franz-type diffusion cells can be divided into side-by-side or upright cells, depending on the orientation of the skin in the diffusion cell. Side-by-side static diffusion cells are seldom used in studies of skin permeation nowadays due to skin damage caused by complete immersion of both SC and dermis in donor solution and receptor media and stirring the solutions at the same time. An overestimated penetration profile can also occur due to excessive and prolonged hydration of the SC through the donor compartment. An upright diffusion cell implies clamping the skin between a receptor compartment containing the receptor media while the SC side faces the open donor compartment, imitating the typical environment to which it is usually exposed [207,208]. When measuring the skin permeation in a Franz cell, attention should be paid to a possible loss of skin tension and elasticity, which can result in over-penetration of the MNs into the dermal tissue. To preserve the original biomechanical properties of the skin, in vitro MN permeation studies can be conducted at full skin thickness [209,210].

Bronaugh-type, flow-through cells maintain a sink condition by using a peristaltic pump that ensures a continuous flow of receptor media through the receptor chamber. Such continuous flow beneath the skin imitates the dermal circulation that carries the exogenous compound away from the absorption site. Moreover, these cells can use automatic sampling that can overcome labor-intensive sampling time points while allowing continuous monitoring of the absorption profiles [211]. Despite these advantages, the complexity and high cost of these flow-through cells lead to more frequent use of the Franz-type diffusion cell. In contrast to the conventional method of in vitro Franz cell permeation studies, MNs are typically applied to the skin before the donor compartment is mounted on the receptor compartment [212,213].

#### 5.3.2. In Vivo Permeation Studies

Neither in vitro nor ex vivo models can fully replicate the in vivo conditions and all skin characteristics. In vivo studies are necessary to investigate the absorption, disposition, and permeation of drugs delivered by MNs. Thickness and elasticity of the skin are crucial parameters that must be considered while selecting the appropriate in vivo model. Although there are differences in structural, histological, and morphological characteristics compared to human skin, the pig model is considered to be the most suitable for transdermal delivery studies [214]. Rodents, especially rats, are also suitable for assessing MN performance because they are inexpensive, easy to handle, and available for different disease models, even though their skin is more permeable than human skin [215,216]. Another important factor to consider when developing in vivo models for MN performance assessments is the application site. Different anatomical sites offer a varying extent of permeability and barrier function of the skin. In addition, the thickness and rigidness of the injection site, which can be affected by lymphatic uptake, play an important role in achieving reproducible results. Measuring plasma levels of tested drugs provides information about their transdermal delivery from MNs, while skin extraction provides information about intradermal delivery. In vivo permeation studies can also provide information about the metabolism of delivered drugs [180].

## 6. Regulatory Issues

Even though 3DP TDD systems offer a variety of possibilities for a suitable approach to every individual patient, regulatory requirements for their application are still not precisely defined. MN patches are considered as devices and have to abide by the terms of good manufacturing practice (cGMP), described in the Quality System Regulations (QSR) [164,217]. AM biomedical products require FDA approval [202]. The biomedical industry is currently concentrating on Class I devices, which require less effort to be approved. However, the development of Class II and III devices is continuing, with the approval of some Class II implants [203]. Class III devices are typically granted an initial investigational device exemption, allowing the use of the device exclusively in an FDA-regulated clinical trial to collect necessary safety and efficacy data before market application. These devices are commonly approved with premarket approval. The FDA developed the Humanitarian Use Device (HUD) Program for devices intended for patients with rare life-threatening diseases or conditions, which allows avoiding conventional marketing processes, clinical trials, and requirements for efficacy data and provides faster and cheaper breakthroughs on the market [218].

Quality requirements of the final product, its safe and effective use, biocompatibility, sterilization, and validation of design must be fulfilled before they can be commercially available. The FDA requires the examination of manufacturing process information. Detailed guidelines are given in their published document “Technical Considerations for Additive Manufactured Medical Devices” [219]. Information about building orientation and all steps of the manufacturing process should be presented. Possible anatomical changes caused by patients’ conditions must be considered when registering a specific device for an individual patient as it may become unusable. All chemical changes of material, as well as the products of degradation that may appear during the manufacturing process due to recurring melting and cooling or photopolymerization, must be noted. Additionally, the stability of the drug with different parameters of 3DP processes, such as high temperature, radiation, or physicochemical alterations, must be proven [147]. Source materials for 3DP and appropriate quality control should be evaluated to ensure homogeneous and traceable manufacturing substrate. The refresh rate of recycled-to-virgin powder with a controlled number of allowed cycles and expiration date or routine retesting of recycled materials need to be defined to prevent diminishing the performance, material contamination, and additional complexities with material traceability caused by the usage of a recycled substrate. Quality measures and consistency of 3DP parameters also need to be documented because they significantly affect the physical properties of the final product [218]. Controlled output and consistent production of devices can be achieved by incorporating the same design and quality control strategies utilized in standard manufacturing methods into 3DP. In addition, cGMP and QSR should be implemented throughout the AM processes [7].

## 7. Conclusions

The unprecedented ease with which complex objects are produced and the relatively low price of commercially available 3D printers have given 3D printing significant popularity and has been spoken of as a third industrial revolution. It has the inherent ability to revolutionize conventional pharmaceutical manufacturing by adjusting the size, shape, and release profile of different drug delivery systems, allowing the precise preparation of personalized dosage forms to address individual patient needs. This technology can play a very important role in the manufacture of personalized TDD systems, as evidenced by the growing interest in the 3D printing of MNs. MNs represent next-generation therapeutic systems that may have a notable impact on clinical medicine in the future, but further multidisciplinary research is needed to obtain ideal MN-based point-of-care systems. Having in mind that AM can be used to rapidly prototype different MN designs as well as MN-based TDD, this approach will undoubtedly optimize the effectiveness of these delivery systems and open new horizons for researchers in the field. Even though photopolymerization-based technologies have been the most widely used 3DP technology in the production of MNs, there is also great potential in all other technologies. However, clinical translation and commercial development of 3DP MNs are still a challenge, and a lot of future work is needed, especially in material selection, optimization of printing and post-printing parameters, and drug loading approaches.

Further investigations are needed to find relationships between MN design and printing parameters and their quality and performance. In addition, future studies need to focus on improving printer properties, such as the laser beam in laser-based methods or nozzle features in extrusion-based methods, to develop faster methods with the highest resolution. Standardized evaluation methods and testing protocols for 3DP MNs also need to be further developed as the variety of mechanical equipment used gives divergent results that are sometimes very difficult to compare. Advances in 3D printing techniques for MN production, as well as recent breakthroughs in electronic mechanicals and artificial intelligence, offer enormous potential for the development of TDD systems that would allow patients to self-administer drugs such as vaccines.

## Figures and Tables

**Table 1:** A summary of materials, advantages, and limitations of the main methods of 3D printing for microneedle production.

3D Printing Technology	Material	Power Source	Layer Thickness	Advantages	Limitations	Ref.
Fused deposition modeling (FDM)	A continuous filament of thermoplastic polymers, glass (new), metal (new)	Heat	50–300 μm	Simplicity High speed Low cost (for thermoplastic materials) Ability to create complex, innovative, and customized dosage forms	The high cost (for glass and metal) Weak mechanical properties (delamination due to temperature fluctuations) Limited material Layer-by-layer finish High temperature during the extrusion process (possible API degradation) Lack of biocompatible/biodegradable printable polymers	[25,28,98,99,100,101]
Stereolithography (SLA)	Liquid photopolymer	UV light	10–400 μm	Simplicity Low printing costs Fine spatial resolution High quality (minimum mechanical anisotropy) Complex and customized drug delivery systems (<100 µm) Minimum drug decomposition	Single material Limited mechanical properties Limited availability of biocompatible photopolymerizable polymers Use of UV light to initiate the polymerization (possible API degradation) Potential toxicity Rinsing and the post-curing process is necessary	[25,102,103]
Digital light processing (DLP)	Acrylates, epoxides	UV light	25–100 µm	High resolution High speed Low cost Less affected by oxygen inhibition than SLA Low initial vat volume	Limited mechanical properties Toxicity Need support	[92]
Liquid crystal display (LCD)	UV-curable resins	UV light	50–100 μm	A smaller volume of resin required High resolution Short curing time Low cost	Low precision	[104]
Continuous liquid interface printing (CLIP)	UV-curable resins, acrylates	UV light	50–100 μm	High speed High precision	High cost Low viscosity resin is needed Probable toxicity	[18,101,105,106]
Two-photon polymerization (2PP)	UV-curable resins, acrylates, ceramics	UV light	100 nm–5 μm	High spatial resolution Scaling up Low-cost materials	Low yield of production Low build speed Limited material	[18,107,108,109,110]
Selective laser sintering (SLS)	Thermoplastics, polymer, metal, and ceramics	Laser beam	20–150 μm	No need for support material High resolution (30 μm) and precision High quality High speed No post-curing required	Limited mechanical properties High cost Slow printing High printing temperature Rough surface Wastage of unsintered powder	[28,101,111]
Direct metal laser sintering (DMLS)	Compact fine powder metals and alloys	Laser beam	20–100 μm	Fine accuracy and resolution Good mechanical properties	Support structures required Protective atmosphere required	[100,112,113]
Selective laser melting (SLM)	Metals, alloys	Laser beam	20–100 μm	No need for support material Good mechanical properties	High cost Poor dimensional accuracy and quality	[28,100,101]

**Table 2:** 3D-printed microneedles for transdermal drug delivery.

3D Printing Technology	Needle Type	Shape	Geometric Features	Ref.
FDM	Hollow	Pyramid	Lengths: 800–3000 μm Base sizes: 500–1500 μm Opening diameters: 200–500 μm Spacings: 1500–3000 μm	[119]
	Solid	Tapered cone	Length: 4500 μm Base diameter: 1500 μm Tip diameter: 500 μm Spacings: 250–750 μm	[117]
	Drug-loaded MN	Conical	Length: 800 μm Base diameter: 700 μm Spacing: 1100 μm	[19]
	Solid	Cylindric	Lengths: 1450–2000 μm Widths: 465–600 μm	[32]
	Drug-loaded MN	Cylindric	Lengths: 200–2500 μm Widths: 400–600 μm Tip diameters: 170–220 μm	[26]
SLA	Drug-loaded MN	Cross-shaped	Length: 1000 μm Width: 1000 μm Length of fins: 430 μm	[96]
	Drug-loaded MN	Pyramid and flat spear-shaped	Length: 1000 μm Base dimensions: 1000 × 1000 μm (pyramid), 80 × 1000 μm (spear)	[126]
	Drug-loaded MN	Pyramid and cone	Length: 1000 μm Base dimensions: 1000 × 1000 μm (pyramid), ø 1000 μm (cone)	[33]
	Hollow	Conical, pyramid, and syringe-shaped MNs	Lengths: 700–900 μm Base width: 800 μm Center bore: 600 μm	[64]
	Drug-loaded MN	Cone MNs on a curved and flat patch	Length: 800 μm Base diameter: 400 μm Tip diameter: 100 μm Interspacing: 800 μm	[125]
	Drug-loaded MN	Cone	Length: 900 μm Base diameter: 300 μm Interspacing: 1800 μm	[45]
DLP	Solid	Rectangular pyramid shapes	Lengths: 1000–1250 μm Base dimensions: 500 × 250 μm, 750 × 250 μm Tip dimensions: 90 μm × 30 μm	[130]
	Drug-loaded MN arrays	Tapered cone	Body: a cylindrical base with a length of 700 μm Tip: a conical tip with a length of 300 μm	[124]
	Hydrogel MNs	Cone	Length: 700 μm	[137]
LCD	Hollow	Cone, square pyramid, screw, and triangular pyramid	Length: 1000 μm Interspacing: 3000 μm	[138]
CLIP	Coated	Square pyramidal	Length: 1000 μm Base wide: 333 μm Spacing: 1000 μm	[140]
	Drug-loaded MN	Arrowhead, tiered, and turret MNs	Lengths: 600–1000 μm Width: 400μm (tiered MNs) Length: 1000 μm Width: 500μm (turret MNs)	[139]
TPP	Hollow	Cone and cylinder	Length: 435 μm Base diameter: 100 μm Tip diameter: 35 μm	[148]
	Hollow	Beveled tip	Lengths: 200–400 μm Diameters: 80–120 μm	[143]
	Hollow	Truncated cone-shaped MN arrays	Lengths: 250–300 μm Base radii: 100–187.5 μm Tip radii: 15–20 μm	[144]
	Solid	Ultra-sharp cone MNs	Length: 200 μm Shank radius: 50 μm Tip radius: 0.5 μm	[109]
	Coated MN	Cylindrical, conical, and pyramidal MNs	Length: 6 μm Tip diameter: 630 ± 15 nm	[142]
DMLS	Solid	Cone	Length: 1000 μm Base diameter: 250 μm Interspacing: 1500 μm	[156]
SLM	Hollow	Cylindrical	Length: 1200 μm Tip diameter: 160 μm	[158]

FDM: Fused deposition modeling, SLA: Stereolithography, DLP: Digital light processing, LCD: Liquid crystal display, CLIP: Continuous liquid interface printing, TPP: Two-photon polymerization, DMLS: Direct metal laser sintering, SLM: Selective laser melting.

## Data Availability

Not applicable.
